# Perceptions of Surrogacy Within the Yoruba Socio-Cultural Context of Ado-Ekiti, Nigeria

**DOI:** 10.12688/f1000research.20999.3

**Published:** 2021-02-08

**Authors:** Oluwatobi Joseph Alabi

**Affiliations:** 1 Department of Sociology, University of Johannesburg, Johannesburg, Gauteng, 2006, South Africa

**Keywords:** Surrogacy, reproduction, infertility, culture, religion.

## Abstract

**Background:** Surrogacy might be a reproductive process that brings joy and fulfilment to many but it also brings with it numerous ethical and legal concerns; it raises questions about the fundamental human rights, welfare and wellbeing of women and infants especially within a context where it is barely regulated. This article examines the perception of surrogacy within the Yoruba socio-cultural context in Ado-Ekiti, Nigeria. It brings to the fore various socio-cultural concerns that questions the influence of surrogacy as a reproductive process on womanhood, motherhood and parenthood. It discusses by analysing the narratives of participants how surrogacy process is a dereliction of the sacredness and cultural sanctity of the family system, most especially in an African context.

**Methods:** 15 stakeholders (traditional birth attendants and gynaecologists) were engaged in an in-depth interview to unravel the challenges surrogacy might or is encountering within the socio-cultural context of Ado-Ekiti.

**Results**: There are various social, cultural and religious beliefs that police the reproductive sphere of the Yoruba socio-cultural group, which has grave implications on fertility treatment. These socio-cultural and religious factors do not provide a fertile ground for surrogacy to thrive within the study location. Hence, it is important that the socio-cultural framing of reproduction within this cultural context become receptive to medical reproductive solutions and innovations if at all the processes are to thrive or at least become less stigmatised.

**Conclusions: **The process of surrogacy is very complex and people’s attitude towards the practice is greatly influenced by their culture, religion and social belief systems about what is considered appropriate for procreation. Also, it is important to have clear-cut policy regulating surrogacy and all forms of ARTs in Nigeria, as this will protect women and infants, as well as, ensure that they are not to exposed abuse, commercialization and exploitation.

## Introduction

Infertility is described as a global public health challenge affecting between 13–17 per cent of couples trying to conceive in sub-Saharan Africa, with a higher rate of about 32 per cent in most of the countries sampled in the survey; this is according to the infertility survey conducted in 27 sub-Saharan countries including Nigeria. As the percentage of infertility remains high in most countries in sub-Saharan Africa (32%), the pressure on couples to procreate and find solutions to the often stigmatized status of infertility is always intense and complex. Infertility across most parts of Africa has not been well researched as a vital part of sexual reproductive health, yet its impact can be highly consequential ^1^. Having biological children is highly desirable and the inability to conceive is often demonised, socially stigmatised, could lead to divorce or results in adverse psychological and health effects on the partners involved ^2^. It is also important to understand the gender narratives around infertility in most parts of sub-Saharan Africa; while men and women can both be potentially infertile, women are often blamed and punished for childlessness ^3, 4^. Within this immensely complex socio-cultural milieu that prioritises fertility and links procreation to the completeness of a man or woman, couples are put under pressure to bear children and failing to do so is regarded as an existential failure. Studies show that the pressure to have children has increased risky sexual behaviour among couples in sub-Saharan Africa ^4^.

The search for infertility treatment is a complex terrain to navigate because infertility possesses socio-cultural attributes and challenges at both foundational and experimental levels ^5^. Explanations of the aetiology of infertility differ between healthcare providers, patients and the society in Nigeria ^5^. Western medicine can diagnose infertility biologically and administer clinical recommendations; however, indigenous medicine believes that factors causing infertility could be biological or even supernatural ^5^. This dichotomy in the aetiology of sickness in general and infertility, in particular across Africa, is foundational for the various socio-cultural narratives surrounding reproduction processes in Nigeria. There are various socio-cultural beliefs policing fertility in Nigeria and these beliefs are of grave concern due to the perception of and attitude towards infertility ^2^. The cultural environment is paternalistic and as such, children are highly desired, and parenthood is culturally mandatory ^2^. Any standard below this paternalistic cultural expectation is regarded as cultural deviation and often stigmatised ^2^.

The challenge of infertility in sub-Saharan Africa is debated among researchers, especially because of the complex socio-cultural narratives surrounding reproduction in the region. As such, various reproduction options have been debated and discussed in the literature. Over the years, adoption has been one of the most popular routes taken by couples in Nigeria struggling with bearing children ^6^, however, as practices such as surrogacy become more globally popular, it is crucial to examine popular perceptions about the process and how it fits into the socio-cultural framing of reproduction in Nigeria. Notwithstanding the popularity of adoption, it is a very challenging and complex process to embrace in Nigeria as a result of various stigma and stereotypes ^7^. A previous study among the Yoruba people of Western Nigeria revealed that the success of child adoption process is marred by several socio-cultural factors, some of which include a lack of adequate information about the adoption process ^8, 9^. These limitations have also been discussed as prominent in Eastern Nigeria ^10^. These socio-cultural challenges are not particular to the South-West or Eastern parts of Nigeria alone, as it is important to note that despite the heterogeneous nature of the Nigerian society, a common narrative across these cultures is the premium placed on children, emphasis on parenthood, and the norm that every woman should conceive and carry children to term ^2^. Implicatively, fertility is highly valued across genders and is often seen as a validation of womanhood or manhood.

Surrogacy is a process whereby a third party (woman) of childbearing age carries a pregnancy for a commissioning parent with the intention of relinquishing the baby after birth, usually enforced by a contractual agreement between the parties involved ^11^. The United Nations Special Rapporteur defined surrogacy as “a form of third-party reproductive practice in which intending parents(s) contracts a surrogate mother to give birth to a child ^12^.” While the birth of baby Louise Brown in 1978 (the first baby conceived through *in vitro* fertilisation in England) rekindled the hope of many childless couples across the world on the possibility of having children that are genetically connected to them ^13^, it must be noted that surrogacy is not necessarily a type of ART but rather a reproductive process that relies on another person (a woman) to carry a child to term. ART has become very popular for treating infertility the world over, but with impeding challenges especially in developing nations. Assisted Reproductive Technology (ART) in general is basseted with numerous challenges in Nigeria and these include, limited reproductive centres; highly priced charges to seek reproductive help as most of the reproductive centres are operated by private organisations that are profit driven ^14^; and, low success rates ^8^. Even though there are many options for infertility treatment across the globe, the success of such processes depend on etiological factors, availability of medically advanced diagnostic tools, skills of the attending physician and financial status of the couples involved ^15, 16^. This study recognises and expands on the various challenges debated in literature in Nigeria and furthers the conversations around other fertility treatments in Nigeria, particularly surrogacy.

Surrogacy as a reproductive process is swamped with numerous challenges spanning through parental rights, the fundamental human rights of the child, and women abuse, specifically the exploitation of surrogate mothers, among others. The legal, ethical, and socio-cultural trepidations debated in the practice of surrogacy remains germane in literature. Some of the concerns that have gained popular attention includes that it is imperative to ensure that the rights of the child are not abused irrespective of the terms of the contract/agreement between parties; and, that the idea that the woman’s body could be perceived as just a medium for reproduction or commercialised without recourse to her emotions or the possibility of bonding with the baby through the period of gestation is abusive and must be addressed. As medical tourism becomes expansive and inter-country gestational surrogacy driven by monetary incentives more popular, the fear of exploitation, conflicting legislation and loss of autonomy have become highly debated in medicolegal and bioethical spheres. It becomes even more complex in a country like Nigeria where there is no clear-cut legislation for surrogacy. There are arguments in literature highlighting the complex terrain in which ARTs, surrogacy and other types of insemination operates in Nigeria. While there are no cut-out legislations regulating surrogacy, it is important to note that there are currently bills in Nigeria’s national assembly that will necessitate regulatory policies for practices like surrogacy if passed ^17^. This has created many loopholes within the reproductive space of the country with practices like ‘baby factories’ thriving as illicit fertility clinics while actually exposing babies and women to abuse and exploitation. This study discusses surrogacy within a Yoruba socio-cultural context of Ado-Ekiti, Nigeria. It brings to the fore the various narratives and perceptions of surrogacy by engaging gynaecologists, legal professionals, and traditional birth attendants through interviews.

## Methods

### Study design

This research is qualitative, and its strategy of inquiry is explorative. In utilising this exploratory approach, the researcher, through painstaking interviews, accessed the perceptions of gynaecologists and, traditional birth attendants (TBA) on the practice of surrogacy in Ado-Ekiti, Ekiti State, Nigeria. Exploratory qualitative research allows the researcher to collect data from persons who possess relevant knowledge about a phenomenon and assists in developing a composite description of the essence of their experience for all individuals ^18^.

### Ethical considerations

This study was approved by the University of KwaZulu-Natal ethical clearance Committee (Protocol number: HSS/0705/017M) after due consultation and the gatekeepers' consent letters were provided by Ekiti State Teaching Hospital, Ado-Ekiti, Ekiti State Nigeria.

### Participants

The study population consisted of two categories of participants. These included ten gynaecologists and five TBAs. This study was conducted at Ekiti State Teaching Hospital, Ado-Ekiti, Ekiti State Nigeria.

Considering that surrogacy is not a popular practice in Nigeria, purposive sampling was used to select participants. Ekiti State Teaching Hospital in Ado-Ekiti served as a welcoming host to the researcher and provided access to participants. The Ekiti State Teaching Hospital in Ado-Ekiti is one of the foremost fertility clinics in the South-western part of the country and thus a suitable location for the study.

Informed consent was gained by detailing the purpose of the study and the strategies to ensure the participants’ anonymity and confidentiality were explained to them before the study began, where after they voluntarily signed the consent forms. The informed consent included that the participants could withdraw from the study at any point if they no longer feel comfortable.

### Sampling

Purposive sampling was adopted to recruit participants in the study. Upon receiving ethical clearance from the University of KwaZulu-Natal, the gynaecological centre of Ado Ekiti Teaching Hospital was visited, and appointments were made to interview gynaecologists at their convenience. The department also served as a link to the association of TBAs in Ekiti State.

### Data collection

Face-to-face in-depth interviews were conducted with each participant using a semi-structured interview schedule that contained three sets of questions. An audio recorder and field notes were used to collect the data. The participants had consented to the recording of the interview with an audio recorder on their informed consent form. The first set of questions examined surrogacy within a cultural perspective, the second questioned medical opinions on surrogacy arrangements as it pertains to Nigeria, and the third examined the legal trepidations in the practice of surrogacy. The interview schedule was translated into Yoruba, the most commonly spoken language in Ekiti State, for the benefit of the TBAs who might not be English speakers. Interviews lasted on average of 30–70 minutes.

Not all the participants approached participated in the study. Considering the nature of the medical profession, the timing was a major constraint; several of the gynaecologists cancelled appointments more than twice, and some were finally not interviewed. However, data saturation was a major guide during the process of the fieldwork. The narratives from participants were constantly assessed to identify when new information was derived, and when there were very frequent repetitions data saturation was reached and the fieldwork came to an end.

Audiotapes were transcribed by the researcher and interview transcripts and summaries were checked with participants to ensure that their narratives were captured correctly and not distorted in any way.

### Data analysis

Thematic content analysis was adopted to analyse the field notes and transcripts. Thematic content analysis is an approach to the analysis of documents and texts (which may be printed or visual) that seeks to quantify content in terms of predetermined categories (in this research emerging categories from participants’ narratives) in a systematic and replicable manner ^19^. Transcripts from the interviews were organised into a logically readable format, after which recurrent patterns and conceptual issues were identified and developed into themes that formed the basis for the analysis. Participant numbers and characteristics (age, gender, and job) (see Table 1) were used in reporting the findings of this study to ensure the anonymity of participants as noted in the informed consent.

**Table 1.** Demographic characteristics of participants.

Gynaecologists		
s/n	Gender	Age
1	Male	37
2	Male	46
3	Male	36
4	Male	39
5	Male	45
6	Male	39
7	Male	Adult
8	Male	38
9	Male	40
10	Male	48
Traditional birth attendants		
11	Female	65
12	Female	49
13	Female	58
14	Female	40
15	Female	53

## Results

The dynamic nature of human societies coupled with varying regulatory normative codes across cultural boundaries creates the need to examine the perception of surrogacy within various cultural settings in order to appreciate its dynamic and contextual peculiarities. The imperative questions that steered this study included a need to understand how surrogacy is conceptualised and understood within the Yoruba reproductive frame(s); and a concern to understand the socio-cultural imperatives influencing/shaping the practice especially among the Yoruba people of Ado-Ekiti, Ekiti State, Nigeria. The themes discussed include the social perception of surrogacy; the socio-cultural intricacies of surrogacy and its implication for the normative understanding of a ‘woman’; the gendered nature of infertility and, the effect of surrogacy on the structure of parenthood.

### Social perception of surrogacy

The participants provided varying thoughts on the popularity of surrogacy. Some of these seemed to be influenced by personal beliefs, religious and cultural sentiments, as well as, their knowledge about the practice. A careful examination of the responses revealed complex narratives that further strengthens the debates in literatures that surrogacy brings to the fore various legal, socio-cultural, and ethical concerns. Most of the gynaecologists expressed support for the practice of surrogacy even though they noted that it is a complex terrain to navigate, while TBAs believe it is a practice that negates normative reproductive processes and a concept that could commercialise babies and the woman’s body. Most TBAs strongly drew on religious and cultural beliefs in their discussion of surrogacy as a reproductive process. One of the gynaecologists mentioned that:

*Surrogacy in Nigeria is not a popular practice, it is not that popular because when viewed from a socio-cultural lens, there are lots of beliefs, controversies that surround surrogacy. In most quarters in Nigeria, surrogacy is not popular at all and I will say the popularity and acceptance of the practice are hugely influenced by socio-cultural, legal, and spiritual factors. The legal space has not created platforms that support or regulate the process and the socio-cultural and religious aspects of our society are not in favour of such acts that distort natural order (Gynaecologist, M, 48 years).*

All the gynaecologists interviewed believe that surrogacy will gain prominence with time and as the country continues to transform; however, they also believe that it is still in its infancy and essentially still unpopular at the moment. They also emphasised that the popularity of the process is hampered by socio-cultural beliefs and values regulating reproduction within the socio-cultural context of the Yoruba people of Ado-Ekiti. Notwithstanding, they noted that surrogacy is becoming a thriving venture in major urban settlements like Abuja, Lagos, and Port-Harcourt, even though the legal terrain in which it operates remains unregulated.

The opinions shared by TBAs elaborated on the religious and socio-cultural factors influencing the popularity of surrogacy in Nigeria discussed in the previous section:

*Our prayer is that this act won’t gain prevalence and acceptance in Nigeria, because it is anti-cultural and anti-religion. God has established a process for that and no one or advancement should change it. It is a devilish act championed by the western world and it won’t succeed in our land. How do you expect a woman that did not gestate a child to know the real worth and value of that child? Or how does the child become a true member of the kin when another woman gestated him/her. God forbid, it won’t work here. I have heard people are doing it already, but God will destroy their plans (TBA, F, 58 years).*

All the TBAs shared the same opinion that surrogacy is becoming a social discussion in Nigeria; however, they hoped it would not thrive within the country because it goes against cultural and religious norms for reproduction. The reservations they have for the practice include the fact that the surrogacy process will destroy the entire process of normal reproduction- which spans through the period of pregnancy, bonding, as well as the premium placed on children. The terrain of surrogacy is indeed very complex and currently does not constitute an important aspect of public discussion ^20, 21^. What surrogacy means and the social reception it enjoys differ across cultural and social boundaries. It is quite evident from participants’ narratives that the discussion of surrogacy brings to the fore several concerns that are often influenced by the social, cultural and religious beliefs of the community ^22^.

Surrogacy and its practice is borne out of a desire to have a child ^23^, and contemporary practice thereof is often hinged on a desire to have a child with a biological connection ^24, 25^. Moreover, the process as discussed is complex and could potentially lead to the exploitation and abuse of fundamental human rights, hence it remains a highly debatable topic. For instance, perceptions of surrogacy may also be grounded in descriptions that suggest commercialisation and exploitation of women ^26, 27^. Commercialising a woman’s body by renting her womb and putting a price tag on the children that is a product of this process is highly dehumanising and disregards a fundamental human right.

TBAs in this research described surrogacy as an act that distorts the cultural normative process for reproduction and disregards the role of women. Surrogacy in Yoruba language is translated as *“agbabi odi omo eni”*, meaning ‘contracted pregnancy does not become yours’. The meaning and definition of the process in this language suggests a difference between the surrogate and child, as well as between the commissioning parent and the child, and that in itself is reinforcing the stigma placed on infertility. Hence, surrogacy is perceived as a way of expressing how one woman was unsuccessful in conceiving a child; this role, however, has been taken up by another woman who has the physiological capability to conceive and carry a child to term, without paying attention to the possible feelings that might be involved as the surrogate mother carries the child to term. The process also fragments parenthood and raises serious paternity issues according to these traditional birth attendants. These narratives by TBAs describe surrogacy as a deviating act and even in cases of altruism, the altruism of the surrogate is regarded as going beyond normative boundaries ^28^. The perceptions of surrogacy among this study’s participants highlights the complexities and multiple contestations that characterises the entire process. Some of the socio-cultural, religious and ethical trepidations mentioned in this sections will be discussed further.

### Fragmenting womanhood: socio-cultural intricacies in the process of surrogacy and the understanding of a ‘woman’

Across most African societies, there is a great deal of significance placed upon a woman. They play a significant role in the continuity of the community and the operation of social life and are considered the fabricators of life and the mothers of humankind. As a major player in the personal rituals associated with birth, puberty, and death, the symbolism of these rituals demonstrates the important cultural meanings of womanhood ^29^. There is a connection between fertility, culture, and religion across most African cultures ^30^. While the ability to conceive and carry a child to term is regarded as a gift and partly the workings of a supernatural being, the cultural definition of a woman is her ability to perform the gestational role of carrying a child to term. Across most of African cultures, high fertility is not only a divine reward but evidence of the right behaviour. Among the Chaga of Tanzania, the wife in complying with the divine order has been described in these words: “she corporates with her husband, the ancestors, even God, in creating the child” ^31^. Hence, it can be inferred that fertility is a product of complete obedience to God and the ancestors, and exuding what is considered conforming behaviours. Children are highly desired, and women are accorded a lot of regard for being fertile, usually adjudged on their ability to conceive, carry to term and nurture a child. The birth of a child is celebrated and seen as a sign of divine approval by both living and dead/ancestors. However, infertility is regarded most times as a woman’s problem and evidence of sin and disapproval by both God and the ancestors ^31^. These are extraordinarily strong gendered assumptions that continue to domesticate women, sexualise their bodies, and produces female oppression within the study’s location.

The perceptions shared by the participants are diverse but entrenches the notion that fertility and children are important parts of the African community. A woman’s ability to conceive and carry a child to term defines her and serves as a rite of passage to womanhood. In addition, religion is fundamental to the conception of fertility, while infertility is an aberration that probably results from sin. Notwithstanding, surrogacy is not seen as a solution to infertility but rather as a further disregard for religion and culture. TBAs believe that an infertile couple must continue to pray and seek solutions that will not be unnatural. When most of the participants were asked about how they think the practice of surrogacy will influence the conceptualisation of womanhood, various issues emerged, including:

*Personally, it is against the definition of womanhood and even the surrogate is not thoughtful enough, what will the surrogate tell her own husband after she must have done something like this. Will she say she has not given birth before or what? What if she cannot give birth again? Even if she marries and now have a child through the cultural medium with her husband, would she now refer to this present child as her first seed or the surrogate child? This is disturbing, confusing and against our culture (TBA, F, 53).*

The notion that surrogacy is a reproductive process that encroaches on the pertinent understanding of reproduction and womanhood was shared by all TBAs and in fact they believe that it is not a practice any of the parties involved will be proud to identify with publicly. This is very significant as it brings to context some of the challenges that emanated in the design of this study. The study at its early stage set out to interview couples who have utilised the services of a surrogates but all those identified and contacted through the help of key informants refused to participate for reasons related to labelling and social stigma. The surrogates would also not want to have anything to do with the study. Furthermore, TBAs believe strongly that surrogacy is a negation of acceptable religious and cultural process for reproduction:

*God created the man and woman. There is a plan and process designed by the creator for procreation. God endowed women for reproduction, and he did not sanction surrogacy or at least my own religion does not teach me to do that. The Bible says there shall be non-barren in the land. That there will be children in your loins, so it does affect the definition of womanhood and the whole commandment of being fruitful (TBA, F, 40 years).*
  The responses captured above further reiterate the intersection between religion and culture in the perception of fertility and procreation within this context and across most African cultures. It is an intersection that evidently shapes the perception of surrogacy in Ado-Ekiti, Nigeria. While these beliefs are arguably producing assumptions that seek to police women’s sexuality and reproductive abilities, it does raise fundamental questions about the legality and ethicality of surrogacy and how it affects women and children.

### Gendering infertility: women’s infertility as a consequence of promiscuity and recklessness

The perception that infertility is regarded as evidence of sin or recklessness was discussed by participants. For example, it was noted that infertility may be the result of the reckless lifestyle the woman may have lived as a youth:

*We cannot change the gender of the woman because she can’t carry a child, but it must be that the woman that requires the service of a surrogate must have lived a reckless life as a youth, maybe she must have aborted three to four times before marriage. So, she will later have problems with giving birth, but the husband won’t know. So, she’s still a woman that has no experience or knowledge of what it means to be pregnant and carry the baby to full term. Most times these couples struggling to give birth, especially the women have been promiscuous and reckless, so she’s reaping the fruit of her labour. Instead of going through this ungodly medium, she must cry unto God and ask for forgiveness. It is only God that can give children (TBA, F, 65 years).*

The perception that infertility is a punishment for a perceived sin or recklessness usually associated with women was quite resounding. The gendered nature of infertility is actually an idea that some participants noted to have shaped the perception of fertility in Nigeria:

*Infertility within our own cultural setting is seen as resulting from factors beyond biology, that is why you see that couples that are struggling to conceive are always told to pray. Even as medical professionals we tell our patients to pray. It is who we are: we are a very religious people. It is just unfortunate that in most cases, childlessness is always seen as a woman’s problem. (Gynaecologist, M, adult).*

The gendered nature of infertility from the response above is quite popular in Nigeria and cuts across class, gender and social status. Equating a woman’s ability to birth a child to her attainment of womanhood is also a common narrative that further stigmatises women. One of the participants noted that:

*Womanhood within our culture is defined in terms of a woman’s ability to get pregnant and carry a child to term. So, a woman that adopts the option of surrogacy will not have an opportunity of giving birth by herself and that will question her womanhood, a lot of people will see her as not being woman enough because she has not been seen to be pregnant. People attach being able to get pregnant as a mark of womanhood, so surrogacy will affect womanhood negatively, especially where people place a lot of emphasis on a woman’s ability to get pregnant, gestate and carry the child to term (Gynaecologist, M, 45 years).*

These narratives suggest that without gestating and carrying a foetus to term, it is culturally believed that the woman is incomplete and has failed in her role, and as such transferred this responsibility to another. ‘Renting’ a womb (surrogacy), as discussed in the literature ^32, 33^, is seen in this context as a cultural anomaly and misnomer. A process that fragments womanhood and commercialises reproduction. Notwithstanding, some participants still believe there are some positives to altruistic surrogacy:

*I have discussed with a lot of people outside my profession and they believe without any doubt that surrogacy taint the cultural as well as religious processes for reproduction but personally I think it is just part of the things we have to accept as the world becomes more globalized, industrialised and medical technology advances. While commercial surrogacy is another debate entirely, I think it is important we also give some attention to the fact that through a process like this come couples who cannot have a child through the natural means can still have a child that is genetically connected to them even though they might have to consult the services of another woman. That I think is a positive that we cannot overlook. (Gynaecologist, M, adult).*

The participant above noted that the failure of a woman to fulfil her cultural role as a woman has essentially created a gap that another women will fill, either for altruistic reasons, e.g. helping the woman fulfil her social expectations of becoming a mother, or for financial gain inherent in the practice of commercial surrogacy. Interestingly, the participants believes commercial surrogacy is complex and requires a more critical discussion. Moreover, findings from this study suggests that surrogacy as it is practiced is new to Nigeria but might start to become entrenched basically because of the premium placed on having children. One participant stated that while the practice negates the normative ways of procreation, it also fulfils a cultural goal of helping other infertile couples build a family:

*In my own view, how it affects the definition of womanhood is; it depends on how you look at it, especially in an African setting where it is believed that if a woman does not bear a child then the woman is not fulfilled. Interestingly, now we have women coming out to help women in the actualisation of their desire to have a baby, so I think this has really gone a long way and has put a smile on the faces of lots of females around the world and it as really makes womanhood more appreciated than what you can ever think of (Gynaecologist, M, 39 years).*

Another participant believes that surrogacy provides support for arguments that parenthood is not just biological but could be social or by assuming the responsibility of raising another person’s child. One of the participants noted that:

*Surrogacy redefines the whole essence of the family and parenthood. It makes us think of a woman beyond just bearing children. It reminds us there is more to a woman than just bearing children because at the end of the day even if there is a problem with the man, everybody stigmatises the woman in a childless union. It means a woman can be a woman without necessarily bearing a child (Legal professional, M, 34 years).*

Further reiterating this line of thought, another participant noted that:

*Hmmm…. Well the definition of womanhood generally around here is complex because in the real sense being a mother is not about giving birth like we also say being a father is not about fathering a child, but the roles and responsibilities assumed. However, we find out that being a woman has been defined with being able to conceive in the African sense, so surrogacy thus affects this definition of womanhood. Not being able to conceive is more or less like you are less than a woman, in fact, I have seen where people have written that if you give birth through caesarean operation (CS) then you are not a true woman. So, I can imagine that in that circle giving birth through a surrogate tampers with the traditional definition of womanhood, however, the world is evolving and people are defining motherhood and fatherhood by roles and responsibilities assumed in the life of a child (Legal professional, F, adult.)*

While some participants discussed surrogacy from a very positive stand by focussing on the fact that it helps individuals fulfil the normative social expectations of raising a family where children are regarded as a premium, but it has failed to bring into context the exploitation, complexities in parentage and other ethical issues inherent in the practice. The conflict between agency and protecting the rights of the child that will be a product of this process, as well as some of the women who are often caught in a complex web of unequal power relations, makes the terrain very complicated to navigate. It also reemphasises the need for a comprehensive reproductive legislation in Nigeria that will protect women and children from all forms of exploitation and abuse.

## Discussion

This study brings to the fore sacrosanct socio-cultural, religious, ethical and legal musings influencing the perception of surrogacy in Nigeria. The study revealed that even though surrogacy appears to be practiced in Nigeria, it is hidden and unpopular. While surrogacy might be glorious news for couples struggling to have children where natural conception is impossible, it is raises various concerns because of its potential to exploit women, commercialise their bodies and disregard the fundamental rights that should protect the child ^21^.

Some of the socio-cultural factors strongly influencing the perception of surrogacy includes the gendered nature of infertility where women are often seen as problematic partners when couples are struggling to conceive. This perception is accompanied by various stigmas that label, diminish and only recognise the reproductive abilities of women. For example, in the present study, the ability of a woman to gestate is attached to her completeness or her attainment of womanhood, because when she fails to fulfil this social expectation, she becomes stigmatised and considered less a woman. This attitude will discourage women from seeking fertility treatment and may lead to couples with fertility challenges becoming secretive about their medical condition. Another stigma associated with infertility is that women having challenges conceiving are believed to have been promiscuous and reckless in youth in some cases. It is important to challenge gendered notions of infertility because it is evident from the narratives shared by participants that when issues of infertility are discussed, women often occupy the centre of the discussion and very little is discussed regarding male infertility. Most of the participants believe that surrogacy is a process that distorts normative socio-cultural definitions of a woman, and commercialises children and the entire process of reproduction. Notwithstanding some other participants are of the opinion that it helps infertile couples achieve their aim of raising a family with the help of another - a surrogate. 

The findings of this study were limited by time and resources. The focus was on one of the fertility clinics at the Ekiti-State Teaching Hospital, and as such does not provide sufficient data for generalising the findings. However, it provides a detailed empirical framework for examining the common perception about surrogacy in Nigeria.

## Conclusions

This study has unpacked the various socio-cultural and religious trepidations in the practice of surrogacy in Ado-Ekiti, Ekiti State, Nigeria. Through painstaking interviews with gynaecologists, legal practitioners and traditional birth attendants, the study unravelled that there are several socio-cultural and religious factors policing the reproductive sphere in Nigeria that makes it difficult for surrogacy to thrive. The overarching influence of socio-cultural beliefs on issues like infertility makes it extremely difficult for processes like surrogacy, considered unnatural, to thrive. Some of the essential findings from this study include that infertility is highly gendered and women often occupy the centre of discussions around infertility. It became evident from the findings that there is a socio-cultural as well as a religious lens through which women are viewed, and this lens does not condone surrogacy. Some of the crucial factors referenced while explaining what it means to be a woman include gestation and carrying a child to term. It is believed that the period of gestation builds a special bond between the woman and the child, and this is important for kinship formation. Interestingly, the lack of specific legislations regulating surrogacy in Nigeria makes the process uniquely challenging and exposes women and infants to potential abuse and exploitation. This legislative lacuna has also fuelled the illicit trade of baby factories that have become quite popular in Nigeria. While the socio-cultural space within the context of this research does not provide an enabling environment for surrogacy to thrive, the premium placed on fertility and bearing children provides an indirect demand for illicit baby factories to continue to grow. There is a need for an attitudinal change in the perception of infertility in Nigeria to reduce stigma and discrimination. Legislative gap in Nigeria has played significant role in the growth of baby factories, hence, the abuse and exploitation of women and babies. As such, it is important to effectively legislate surrogacy and provide a broad legislative framework for reproduction so as to protect women, children and babies from abuse, exploitation and commercialization.

## Data availability

### Underlying data

Figshare: Surrogacy Transcript. figshare. Dataset. https://doi.org/10.6084/m9.figshare.11120891.v1 ^34^.

Data are available under the terms of the Creative Commons Attribution 4.0 International license (CC-BY 4.0).

### Extended data

Figshare: Interview schedule, https://doi.org/10.6084/m9.figshare.10264904.v1 ^35^.

Data are available under the terms of the Creative Commons Zero “No rights reserved” data waiver (CC0 1.0 Public domain dedication).
