# Supplementary material for: Perceptions of Surrogacy Within the Yoruba Socio-Cultural Context of Ado-Ekiti, Nigeria
Source: F1000Res. 2021 Feb 8;9:103. Originally published 2020 Feb 11. [Version 3] doi: 10.12688/f1000research.20999.3 (PMC7921890; doi:10.12688/f1000research.20999.3)
Supplement: Peer review history

# Peer review: Reviewer response for version 3

**DOI:** 10.5256/f1000research.54257.r79077

**Published:** 2021-03-01

**Authors:** Olufemi A. Fawole

**Affiliations:** 1 Department of Sociology, University of Ilorin, Ilorin, Nigeria

I have no further comments to make. The updated version reflects a significant improvement.

Is the work clearly and accurately presented and does it cite the current literature?

No

If applicable, is the statistical analysis and its interpretation appropriate?

Not applicable

Are all the source data underlying the results available to ensure full reproducibility?

Yes

Is the study design appropriate and is the work technically sound?

Yes

Are the conclusions drawn adequately supported by the results?

No

Are sufficient details of methods and analysis provided to allow replication by others?

Yes

Reviewer Expertise:

Marriage and the Family

I confirm that I have read this submission and believe that I have an appropriate level of expertise to confirm that it is of an acceptable scientific standard.

# Peer review: Reviewer response for version 2

**DOI:** 10.5256/f1000research.29719.r75103

**Published:** 2020-12-03

**Authors:** Olufemi A. Fawole

**Affiliations:** 1 Department of Sociology, University of Ilorin, Ilorin, Nigeria

The article seems to concentrate more on a particular social group or ethnic group - Yoruba people than on the entire country of Nigeria. Perhaps this will effect a modification of the title of the paper to suit this. Therefore, the topic could be "Perceptions of Surrogacy Within the Socio-Cultural Context of Yoruba People in Nigeria" or Perceptions of Surrogacy among the Yoruba People of Nigeria". The emphasis is on the Yoruba People as these form the basis of the study. The before are simply suggestions to guide the author on the formation of the topic, and need not necessarily be attended to strictly.

 The author may wish to make a comparison between the three major ethnic groups - Yoruba, Hausa and Igbo. But only if it will not be out of the focus of the paper.  The author could also attempt to have a look at surrogacy among the cultural group in pre and post colonial times. Having a look at surrogacy in traditional times versus contemporary reasoning could make an interesting contribution to the paper.. 

 The paper concentrated on Ekiti State. The question "Why?" now comes to mind. It is well known that the Yorubas cover the entire South-Western part of Nigeria. What then has become of the core areas such as Oyo and Osun states? Unless the title would now be further limited to "....among the Ekiti People of Nigeria. This will justify why the author is focusing on that region alone. This might be a bit problematic, but could the author identify a few of some of such people who have attempted surrogacy and interview them? Having data from those who actually practiced surrogacy would also help in adding to current literature.

 Under "Discussion", 2nd paragraph, line 7, the author stated: " *Another stigma associated with infertility is that women having challenges conceiving are believed to have been promiscuous and reckless in their youth...*" Was this the perception of those interviewed, or the actual report of such women? this is why having data from women who have practiced surrogacy would go a long way in this study.

 The same issue arises here - "b *ecause it is evident from the narratives shared by participants that when issues of infertility are discussed, women often occupy the centre of the discussion and very little is discussed regarding male infertility.*" (Same paragraph, lines 9 & 10. Having those "narratives" from the women involved would make the study more profound. 

 Another question that comes to mind, is surrogacy  seen as an alternative practice to having a child, or a "way out" of the problem of childlessness? To what extent are the people embracing surrogacy as either an alternative method of child bearing or a solution to childlessness? It is possible that some have seen the practice as an alternative, to save them the "troubles" of child bearing. 

 The author could try these references, to see if they will help:

 Ajayi, A. B & Ajayi, V. (2018) Gestational Surrogacy in Nigeria.

 Adelakun, O. S. (2019) The concept of surrogacy in Nigeria: Issues, prospects and challenges.

 Author should work on their references to ensure they are complete.

Is the work clearly and accurately presented and does it cite the current literature?

No

If applicable, is the statistical analysis and its interpretation appropriate?

Not applicable

Are all the source data underlying the results available to ensure full reproducibility?

Yes

Is the study design appropriate and is the work technically sound?

Yes

Are the conclusions drawn adequately supported by the results?

No

Are sufficient details of methods and analysis provided to allow replication by others?

Yes

Reviewer Expertise:

Marriage and the Family

I confirm that I have read this submission and believe that I have an appropriate level of expertise to confirm that it is of an acceptable scientific standard, however I have significant reservations, as outlined above.

# Response

**Published:** 2021-01-26

**Authors:** Oluwatobi Alabi

**Affiliations:** University of Johannesburg, South Africa

Thank you very much for the very constructive recommendations. Most of your recommendations have been put into consideration as you will see in the most recent version of this article. 

 Moreover, I will like to highlight the following to provide context and clarification. The aim of the study was simply to present the contemporary perception of surrogacy within the socio-cultural context of this location. While the review of scholarly literatures presented was intended to give context to the study and highlight the reasons why it has become important to critically examine reasons why surrogacy might not be a popular reproduction option in Nigeria. As an empirical investigation, it is also very important to stay within focus because of the purpose and the limitation of time and resources. For emphasis and in line with the guideline of a qualitative design, it is important to stay within reasonable context since the purpose of the study is not to generalize but provide indepth knowledge of a specific problem within a certain context. The aim of this study is to understand the socio-cultural context of surrogacy within Ado-Ekiti and the topic has been further clarified.  

 The narratives discussed emanated from the responses of participants and the categories of participants in this study have been clearly explained. The study in some instances also indicated that reasons to why surrogates and their clients have become a ‘hard to reach population’ in Nigeria and this is closely related to the social stigma accompanying medical reproductive options. Clarifications have been made all through the article to ensure that surrogacy is discussed not as an option to reproduction but as one of the medical alternatives to treating infertility or rather a reproduction process.    

 Thank you again and I sincerely hope you enjoy reading the latest version of this article.

# Peer review: Reviewer response for version 1

**DOI:** 10.5256/f1000research.23108.r60862

**Published:** 2020-04-07

**Authors:** George Mbara

**Affiliations:** 1 Discipline of Criminology and Forensic Studies, University of KwaZulu Natal, Durban, South Africa

The article presents surrogacy as an aspect of assisted reproductive technology (ART) and explores the challenges inhibiting the acceptance of the practice among Nigerians. The study collected its data from a particular region of the country (South West) and thus, makes generalisations on the findings. Most of the respondents were collected from one ethnic group (the Yorubas) and mostly professional gynaecologists. The findings of the study were erroneously generalised as representing the entire country. 

 Nigeria is not a homogeneous country as presented in the title of this investigation. The country has over two hundred and fifty (250) ethnic nationalities domiciled within its borders ^1^. This diversity transcends religion, culture and socio-political inclinations. Years before the 15th century, most of the ethnic groups that make up present-day Nigeria can all trace their origins to different states. Among the early states were the Benin Kingdom, the Yoruba Kingdoms, Hausa States, Nupe, and the Igbo-ukwu. Countless small states existed around the Lake Chad region which was later swallowed or dispersed during the expansion of Kanem to the North East of Lake Chad (Mbara, 2018:12). Historically, these groups were differentiated based on their socio-political organization. The Yorubas, Hausas and the Binis had a centralized administrative system while the Igbos of the East have variously been described as a “stateless” society because unlike the Hausa, Yoruba and Benin Kingdoms, they did not have a central political institution. Further, the indigenous people that inhabited the country today were profoundly divided by their historical experiences, culture, political development, and religion.

 Thus, a topic like this should look at surrogacy based on the teachings and practices of a particular culture or religion. Investigating surrogacy in ‘Nigeria’ as a whole makes the study problematic given the fact that the three major tribes (Igbo, Yoruba and Hausa), for instance, have very diversified cultural and even religious beliefs/inclinations which deeply impact their social existence. The author alludes to this when he declared, “What surrogacy means and the social reception it enjoys differ across cultural and social boundaries” (p.5). For this reason, this study will best suit a particular ethnic nationality or religion in Nigeria. Just as their ethnicity and religion differ, so to their belief systems are very divergent. The influence of Western education on these ethnic nationalities intensified this division among the people leaving some very conservative (the North) and others more progressive (the South) at varying degrees. This makes surrogacy a very contentious and subjective issue.

 Consequently, the best title for this work should be “A qualitative investigation of surrogacy as a panacea for infertility among the Yoruba people of Nigeria” given the setting of the interviews coupled with the fact that “The interview schedule was translated into Yoruba, the local language of this Western region of Nigeria for the benefit of the TBAs who might not be English speakers” (p.4). With the vast size of Nigeria and the heterogeneous nature of the country, getting most of your sample population from Ekiti State will surely not give you enough grounds to caption the findings as Nigeria. Buttressing this view, Alabi (2020:5) in his results submits, “The dynamic nature of human societies coupled with varying regulatory normative codes across cultural boundaries creates the need to examine the perception of surrogacy within various cultural settings to appreciate the differences and similarities that are obtainable in the growth of the practice as a core ART process”. Thus, he cannot lump over 250 ethnic nationalities in Nigeria and discuss them as a single culture. A little search will reveal that surrogacy has been in practice in many Nigerian societies and in different forms.

 Furthermore, for a more holistic view of the subject matter of this investigation, the stakeholders for the interview should have included a Christian, a Muslim and an adherent of the African Traditional Religion (ATR). This is because Nigerians are a deeply religious people and their religion impacts their culture, lifestyles and value systems. Likewise, some custodians of culture like traditional rulers from the major ethnic groups should also constitute the stakeholders. However, the problem of ethnic minorities and the fear of domination will make it more difficult to select the cultural groups that will participate in the investigation. Thereby justifying the need to narrow down the investigation to an ethnic/religious group in the country. If the investigation is done based on the above recommendation, the author will find very diversified views on the subject matter in Nigeria.

 Surrogacy, as presented in this text (ART or fertility treatment), is quite alien to most Nigerian cultures and so, the scholar presents it as a taboo in the country as evidenced in his findings. In the first place, ART is the medical procedures used in treating the problem of infertility. Such procedures include, in vitro fertilization, cryopreservation of gametes or embryos, intracytoplasmic sperm injection, among others. I align my thoughts with the first reviewer who declared, “Surrogacy is not an ART nor a ‘fertility treatment’ (p. 3), as the insemination in some countries is also achieved with coitus (there are documented cases in Mexico and China)” (Danna, 2020:10). The relationship between surrogacy as an ART as presented in the text is shrouded in ambiguities thus leaving room for multiple interpretations. Surrogacy (renting a womb, see pages 7 & 8) has been in practice in different forms in many Nigerian cultures. In some cases, the problem of infertility is solved by the man consenting to a family member impregnating his wife on his behalf. The “sperm donor” lays no claim to the child. The impotent man plays the father figure to the child who bears his name. In other cases, it was the barren wife in a marriage that took a younger wife for herself. Correspondingly, when a man dies childless, a family member can sleep with the woman (his wife) to produce an heir for the deceased (See the Igbos of Eastern Nigeria, the Ibibios of South-south Nigeria). In these cultures, these practices are often altruistic in nature.

 In particular, among the Igbos of Eastern Nigeria, surrogacy has been in practice in various forms like the woman-woman marriage. Commenting on this practice, Aliyu (2018: para 3) maintains:

 In these various societies, the wives other women married were ladies-in-waiting, surrogate mothers, and daughters-in-law… Woman-to-woman marriage allowed for greater freedom of sexuality for the wives, they could have boyfriends, anonymous men whose only duty was to supply sperm, Achebe calls them “male sperm donors”, and this was socially accepted. Any child the wives had was taken care of by their female husband and carried her name, and this was legitimate in the eyes of society.

 Woman-to-woman marriage practice did not include any sexual contact between the female husband and her wife. This was not "lesbianism," since none of the women who married other women was drawn to other women either romantically or sexually. Children were the prime attraction, every woman who turned into a female husband only wanted a child considered legitimate in the eyes of society. In the same vein, Nwoko (2012) gives other examples of surrogacy among the Mbaise Igbo where a family's female children jointly paid the pride price of a younger lady after the death of their father in the name of their eldest sister, so that the new bride could procreate and raise male children to maintain the lineage of the family. Essentially, a well-behaved young man was chosen by the young bride from the kindred, but in most cases, the bedmate was chosen from the female husband's blood relationship. This had significant implications as it was aimed at maintaining the blood tie of that particular family and to prevent the young bride from polluting the family by raising children fathered by persons with strange ailments, thieves or miscreants.

 To actualize the essence of the marriage, Nwoko (2012:76) adds:

 The female husband remained the sociological father of any resulting offspring. The children belonged to the lineage of her father, not to their biological father. Consequently, she played the role of the father, provider, protector and indeed all the functions and responsibilities enshrined in the patriarchal concept which included physical protection of the family and its territory, the male economic sphere, the spiritual sphere, the social sphere, etc.

 Surrogacy in the context of ‘renting’ a womb is still a cultural anomaly in most Nigerian cultures, but as an altruistic act, it has been around over the years. 

 Moreover, how can surrogacy be a panacea for infertility in Nigeria? The simple meaning of panacea is “cure-all”. Surrogacy can be an option, not a panacea since there are multiple factors responsible for infertility, some are more complex than others. Various traditional treatments are available to infertile couples in Nigeria. Thus, surrogacy cannot be a panacea as the author implies.

 Additionally, in the abstract, 15 stakeholders were stated by the author as respondents, but in the methodology, 10 gynaecologists, 5 TBAs and 5 Legal professionals making a total of 20 respondents. Please reconcile the numbers and justify the sample size especially the 10 gynaecologists. This investigation is more of a social phenomenon than medical and should have covered custodians of some traditions/cultures. Gynaecologists can provide us with some scientific aspects of fertility treatment but not surrogacy as a cultural practice. Why were social workers not interviewed?

 Besides, in the data collection under methodology, the scholar declares, “The first set of questions examined surrogacy within a cultural perspective” (p.4). Who provided this information? In my opinion, the traditional rulers or elders of various cultural groups should be in the best position to provide the necessary information here. They are the custodians of the various cultures, not the TBAs. If due diligence was carried out here, the findings would have been remarkably different from what we have here.

 ‘Interviews lasted on average of 30 minutes.’ Unnecessary repetitions.

 Moreover, in p.5, the author states, ‘The opinions shared by TBAs were grounded in religious and cultural beliefs, leading to opposition to its practice”. This response was perhaps predicated on the false assumption that surrogacy is an “alternative to infertility”. Besides, one can ask, what religion and what culture in Nigeria? This gives room for ambiguities and subjective interpretations. Even when it comes to religions like Christianity in Nigeria, some sects/denominations hold more radical or conservative approach to issues surrounding modern-day surrogacy (Renting a womb with pecuniary incentives). For example, the Catholics and Jehovah Witnesses will surely diverge in their views. The same applies to the Deeper lifers (radicals) and say a Baptist Christian (conservatives). Even in ATRs, the various cultures will have varying degrees of acceptance or abhorrence for surrogacy. There are also individuals in Nigeria who, as a result of Westernisation, have accepted this practice. So many young couples in Nigeria now see surrogacy as a viable option for procreation.

 In p.6, the scholar declares, “However, infertility is regarded most times as a woman’s problem and evidence of sin and disapproval by both God and ancestors”. The influence of Christianity (Catholicism in particular) is fast changing this view among some Nigerian Catholics as Canon 1084.3 clearly states that sterility does not forbid nor invalidate a marriage (when there has been no form of deceit). A childless marriage is valid, and companionship is equally very important in marriages. In preparatory classes, intending couples are exposed to this reality. While a childless couple keeps seeking a remedy to their condition, they are made to understand that it does not invalidate/nullify their union. For this reason, a marriage contracted under the church cannot be dissolved for the reason of childlessness. This explains why you find pockets of couples who have been in the union for decades despite been childless. This does not, however, negate the fact that fertility is still a very integral part of the people/lay faithful.

 This investigation should look at surrogacy as a “viable” option in the case of infertility instead of a ‘panacea’ since it is an exploratory study which discovered that the practice is still very alien to the people and unacceptable. The title as it is gives the impression that surrogacy was discovered to be the ‘solution’ instead of an ‘alternative’. If I am right, the author seeks to sell the idea of surrogacy as a viable option in the case of infertility, not a total solution. The word ‘panacea’ denies other viable options available like IVF, adoption, polygamy or cases where a relative impregnating the wife of an impotent brother to preserve the bloodline, or where a woman marries another woman with the sole purpose of procreation.

 The study pointed out the factors militating against the adoption of surrogacy as a panacea to infertility in Nigeria but failed to establish the need for surrogacy in the country thereby making the title a misnomer. To declare surrogacy a panacea requires that other alternatives were investigated and found wanting, thus necessitating the adoption of surrogacy as a “cure-all” remedy.

 Consider women who give birth through a caesarean section? Some Africans (Nigerians) still do not see them as complete women since they have not felt the pangs of birth.

 Lastly, some typographical/grammatical errors were observed in the work – articles were either omitted or wrongly used. For example, subheading (p. 7): “Surrogacy reorganizing the essence of parenthood in Nigeria” (Add colon or dash after ‘Surrogacy’), “While surrogacy might be glorious news…” (p.7. rework), “and it often seen” (p.3. Rework).

Is the work clearly and accurately presented and does it cite the current literature?

No

If applicable, is the statistical analysis and its interpretation appropriate?

Not applicable

Are all the source data underlying the results available to ensure full reproducibility?

Yes

Is the study design appropriate and is the work technically sound?

Yes

Are the conclusions drawn adequately supported by the results?

No

Are sufficient details of methods and analysis provided to allow replication by others?

Yes

Reviewer Expertise:

Political Science, International relations, cyber security, security studies, African philosophy and ethics, public governance and climate change.

I confirm that I have read this submission and believe that I have an appropriate level of expertise to confirm that it is of an acceptable scientific standard, however I have significant reservations, as outlined above.

## References

[1] Nigeria Fact Sheet. *United States Embassy in Nigeria*.2019;22(4) : Reference source

[2] : Woman-woman marriage in Pre-Colonial Igboland. *The Rustin Times*.2018; Reference source

[3] : Nigeria’s Quest For A Permanent Seat At The United NationsSecurity Council: An Appraisal. *IOSR Journal Of Humanities And Social Science*.2017;22(4) : Reference source

[4] : Husbands in Igbo Land: Southeast Nigeria. *The Journal of Pan African Studies*.2012;5:

# Response

**Published:** 2020-09-17

**Authors:** Oluwatobi Alabi

**Affiliations:** University of Johannesburg, South Africa

Hi George, 

 Thank you for your very insightful comments on this article. I find them very useful for the review and the recommendations in most of the case helped in the production of the new version.

 However, unlike you implied in some instances, the article never assumed to generalize its findings to the entire Nigeria and in no instance was Nigeria as a country presented as the study location. While the topic might have Nigeria named which was a mix up during the editorial process with the journal, the methodology gives the reader a detailed description of the specific study location in Nigeria. Likewise, as a qualitative study, it aim was not to generalize findings but provide a more detailed understanding of surrogacy within the study location. 

 Also, the article does not intend or aim to make a case for/against the practice of surrogacy in Nigeria. Rather, it seeks to investigate how various socio-cultural and religious factors influencing reproduction in Nigeria and south-west Nigeria in particular shape the perception of surrogacy within the studied population. 

 Thank you again for your insightful comments.

# Peer review: Reviewer response for version 1

**DOI:** 10.5256/f1000research.23108.r59860

**Published:** 2020-02-20

**Authors:** Daniela Danna

**Affiliations:** 1 Department of Social and Political Science, University of Milan, Milan, Italy

The proposed article is based on personal interviews with professionals in health and law on the subject of surrogacy. The author takes a clear stand for “modernization”, where he considers surrogacy an ART. He presents in a favorable light the positive attitudes towards surrogacy by legal experts and gynecologists (mostly male) and in a negative light the objections expressed by traditional birth attendants (all female).

 The objective of the article is to identify cultural obstacles to the normalization of this practice, as the author considers it a panacea for infertility, therefore positive, progressive, good.

 There are multiple problems with his view, the principal one is the blurring of what happens in surrogacy and the imprecision in its description, that is missing its concrete aspects. (This blurring is surprising, considering that my own contribution Contract children, a book exactly describing at the global level the phenomenon and its legal and ethical requisites, is listed in the bibliography. I have since published another article that might be of help to the author: “The subrogation of motherhood: a juridical institution putting kinship on the market”, in Salute e società, n. 2, 2019, pp. 44-59 ^1^). Instead of providing a description, the author defines surrogacy as an ART or an “alternative to infertility” (p. 5), and the whole paper rests on this faulty premise.

 Surrogacy is not an ART nor a “fertility treatment” (p. 3), as the insemination in some countries is also achieved with coitus (there are documented cases in Mexico and China). It is instead the motherhood of a woman that will not be considered the legal mother – by deception of the authorities or by changes in the filiation laws. It is essentially a third way to become legal parents besides birthing (with or without recognition by the natural father, a recognition that happens automatically when the mother is married) and adoption. Therefore juridical language should be employed, as it is a juridical institution: the subrogation of motherhood. But out of simplicity I will also call this practice “surrogacy”.

 The mother, that is – in objective language – the woman who has given birth, renounces by contract to her right to the baby (called in law the principle of mater semper certa) in exchange for money, often called “reimbursement”. Where are those “cases of altruism” that the author claims to stand on a par with the commercial version (p. 5)?

 Illegal surrogacy happens, and it is just a practice where the delivery of the baby to third persons is achieved by payment or by illegal force, but when it is regulated by law – despite usually taking place in clinics – is not at all a medical practice, but a pregnancy not essentially different from other pregnancies. The IVF can or cannot take place, and the insemination can happen outside clinics. The fact that surrogacy is clinically managed does not pertain to the core of the practice, which is the legal possibility to sell filiation by the mother to the prospective, and paying, parent(s).

 It is therefore wrong to state in the abstract that, before surrogacy, adoption was “the most common alternative” to legally become a mother, as adoption was the only alternative to birthing a child.

 The major problem with the aseptic definition of surrogacy as an ART – that is, as if it were technology – is that this definition takes for granted that the woman who gives birth will renounce her baby, will renounce her right to legally recognize her baby. But this is not inherent in the practice, which is merely based on a promise. The certainty will only come with legally binding documents: a contract that is currently invalid in most countries. She will surely give up the baby only if she is bound by a contract that is deemed legally valid – for this reason is surrogacy a juridical institution. This use of a woman’s body to obtain a son or daughter – I repeat – is in nearly all cases paid for, and the Rapporteur for the U.N. on child trafficking has already declared commercial surrogacy to be trafficking in babies, and – in her last contribution on the subject that I have read – awaited for supporters of “altruistic” surrogacy to demonstrate that such a thing really exist and is not merely a label for the same selling of commissioned newborns. The author apparently ignores the Rapporteur’s conclusion that commercial surrogacy is a violation of the human rights of the newborn, it is an indignity suffered by a mother, and becomes a violation of her human rights, too, when she changes her mind having developed a motherly relationship with her baby but cannot keep him or her because she signed a contract more than nine months before.

 But how can the author ask others about “how surrogacy is conceptualized in Nigeria” while he has a superficial concept of surrogacy as (just) an ART? Why does he cancel out the legal debate, and does not give the reader the fundamental information of how surrogacy is legally defined in Nigeria? And if it does not appear in law (as far as I know this is the case), what are the consequences for people who may have done informal agreements? Have there ever been real cases? There are sources on forcing pregnancy on women already trafficked for the purpose of prostitution – should this not be discussed?

 How can he claim that gynecologists have a “more informed opinion” on surrogacy (p. 5) when his own description of it is blurry and essentially wrong? Other questions that come to my mind pertain to the framing of the interviews that he conducted: Do not gynecologist talk from their own cultural point of view, like the TBAs? What is “scientific” about their opinions on surrogacy? It seems to me that the TBAs are closer to target (see end of the review).

 In general, the interview material could be analysed in more depth and with more quotations.

 The author’s denomination of “modern” for surrogacy is hardly accurate, as it is well know that there are descriptions in the Bible. The author is worried about stigma creation, but what if surrogacy in itself is violence? Violence against the newborn, who is not a tabula rasa but needs his or her mother, and actively looks for her for the nourishment from her breasts? Violence against the numerous women who do not want to give up their babies after birth but are forced to do it by monetary penalties or even by the force of the State (see the cases of Baby M and Melissa Cook, among many others)? Should acts of violence be free from “stigma”? Should not surrogacy be correctly nominated for what it is: an unnecessary separation of the dyad mother-newborn because intermediaries (and doctors and legal experts, as we see in other countries) profit from the plight of infertile couples, suggesting and organizing this trafficking for their own profit? It seems to me that the explanation for the favorable opinions of doctors and legal experts that the author has found, could be grounded in their own prospect of becoming part of these agreements, organize them and profit from them.

 There is a class dimension and an inequality basis in surrogacy that are totally foregone in the proposed article: there is no altruistic surrogacy but women in need that accept to become pregnant and give up their babies to richer couples/singles because they are paid. (Of course there is the question of consciousness, of subjectivity: many women who get paid say that this is not their primary goal, but without money they most certainly would not undergo a pregnancy for others.)

 Therefore there is no “purely altruistic” commitment (very few exceptions apart) as ALL the legislations canceling mater semper certa allow for “reimbursements” that can be conspicuous. Without money, there would be no surrogacy of relevance, just a few cases now and then. But the aim of the author – whether he realizes it or not – is to set up a market for newborns by obscuring what is really taking place in the countries that have introduced surrogacy.

 Another problem with the general framework of the paper is that, even though the author laments the identification of “woman” with “mother”, he basically suggests a method for a woman to become (by means of paying another woman) a mother even if the couple is infertile. Then we are back to square one with the identification woman=mother. A true alternative would be a change in culture that, instead of working for the social acceptance and normalization of rich couples paying poor women for their babies, would work for the social acceptance of women who cannot, or do not want, to become mothers.

 I am no native English speaker, so I cannot judge the overall quality of the language, but I noted that there is sometimes a lack of grammatical concordances between verb and subject, and there might be other errors (as in my own text). I do not understand for example “data saturation”.

 Other minor points: at p. 3 what is “holistic” in ART procedures, that is the artificialization of reproduction, that result in babies being born with more health problems than naturally conceived babies? What is a “foundational and an experimental level” (same page)? Why is infertility considered a particular case of “sickness” (same page), when the organism that is infertile is in fact generally healthy? What is a “visual text” (p. 4)? Should “corporate” at p. 6 not be “cooperate” (interesting slip of the tongue, as the article advocates for a new market in newborns)?

 Finally: at p. 8: surrogacy is not “one of the most progressive ways of addressing infertility”, nor “a desirable fertility alternative”, but buying and selling newborns exploiting the poor women that are forced by need to take up pregnancy as a job (even in the US they sign surrogacy contracts for such goals as paying for the tuitions of their other children). The TBAs might express this and the refusal of such a market in the language of religion, but they are closer to reality than the various experts who stand to gain from the introduction of this juridical institution.

Is the work clearly and accurately presented and does it cite the current literature?

No

If applicable, is the statistical analysis and its interpretation appropriate?

Not applicable

Are all the source data underlying the results available to ensure full reproducibility?

Yes

Is the study design appropriate and is the work technically sound?

Yes

Are the conclusions drawn adequately supported by the results?

No

Are sufficient details of methods and analysis provided to allow replication by others?

Yes

Reviewer Expertise:

NA

I confirm that I have read this submission and believe that I have an appropriate level of expertise to confirm that it is of an acceptable scientific standard, however I have significant reservations, as outlined above.

## References

[1] : The subrogation of motherhood: A judicial institution that puts kinship on the market. *SALUTE E SOCIETÀ*.2019; 10.3280/SES2019-001004 44-59 10.3280/SES2019-001004

# Response

**Published:** 2020-09-17

**Authors:** Oluwatobi Alabi

**Affiliations:** University of Johannesburg, South Africa

Hi Daniela, 

 Thank you for your very insightful comments on this article. I find them very useful for the review and the recommendations in most of the case helped in production of the new version. However, I will like to state emphatically that I stand to benefit nothing from the commercialization of surrogacy in Nigeria like you implied in some instance.

 In fact, the article does not intend or aim to make a case for/against the practice of surrogacy in Nigeria. Rather, it seeks to investigate how various socio-cultural and religious factors influencing reproduction in Nigeria and south-west Nigeria in particular shape the perception of surrogacy within the studied population. 

 Thank you again for your insightful comments.
